# Interleukin-6 inhibition in the management of non-infectious uveitis and beyond

**DOI:** 10.1186/s12348-019-0182-y

**Published:** 2019-09-16

**Authors:** Samendra Karkhur, Murat Hasanreisoglu, Erin Vigil, Muhammad Sohail Halim, Muhammad Hassan, Carlos Plaza, Nam V. Nguyen, Rubbia Afridi, Anh T. Tran, Diana V. Do, Yasir J. Sepah, Quan Dong Nguyen

**Affiliations:** 1 Byers Eye Institute, Spencer Center for Vision Research, Stanford University, 2370 Watson Court, Suite 200, Palo Alto, CA 94303 USA; 2 Department of Ophthalmology, All India Institute of Medical Sciences Bhopal, Bhopal, Madhya Pradesh India; 3 Department of Ophthalmology, School of Medicine, Gazi University, Ankara, Turkey; 4 University of Texas Southwestern Medical Center, Dallas, TX USA; 5 Department of Ophthalmology, Hospital Universitario de León, León, Spain; 6 University of Nebraska, Lincoln, USA

**Keywords:** Uveitis, Interleukin-6, Newer biologics, Non-infectious uveitis, Tocilizumab, Steroid-sparing therapy, Immunomodulatory therapy, Biological therapy, Interleukin-6 inhibition

## Abstract

**Background:**

Uveitis consists of a spectrum of inflammatory disorders characterized by ocular inflammation. The underlying pathophysiology consists of a complex interplay of various inflammatory pathways. Interleukin 6 is an important mediator of inflammation in uveitis and constitutes focus of research toward development of newer biological therapies in the management of non-infectious uveitis.

**Main body:**

Pan-blockade of the inflammatory pathways with steroids is generally the first step in the management of acute non-infectious uveitis. However, long-term therapy with steroids is associated with systemic and ocular side effects, thereby necessitating the need for development of steroid sparing agents. IL-6 is a cytokine produced by various immune cells, in response to molecular patterns and affects multiple inflammatory cells. In particular, IL-6 is involved in differentiation of CD-4 cells into Th-17 cells that have been shown to play a significant role in various immune-mediated diseases such as uveitis. This broad-spectrum immunomodulatory activity makes IL-6 an excellent target for immunomodulatory therapy. Tocilizumab was the first IL-6 inhibitor to demonstrate efficacy in humans. It inhibits IL-6 from binding to both membrane-bound and soluble receptor and can be administered via intravenous (IV) and subcutaneous (SC) routes. It has been FDA approved for treatment of rheumatoid arthritis (RA) and juvenile idiopathic arthritis (JIA). Following the approval in systemic diseases, its efficacy was demonstrated in various uveitis studies including a phase 2 clinical trial (STOP-Uveitis). Overall, tocilizumab has shown a good safety profile with the risk of malignancy consistent with that expected in patients with rheumatoid arthritis. However, tocilizumab therapy has been shown to increase the risk for gastrointestinal perforation and dose-dependent neutropenia. Following the success of tocilizumab, several other agents targeting the IL-6 pathway are in the pipeline. These include sirukumab, siltuximab, olokizumab, clazakizumab, and EBI-031 which target IL-6; Sarilumab and ALX-0061 act on the IL-6 receptor.

**Conclusion:**

Studies have shown that IL-6 inhibitors can be effective in the management of NIU. In addition, the levels of IL-6 are elevated in other ocular vascular diseases such as retinal vein occlusion and diabetic macular edema. The roles of IL-6 inhibition may be broadened in the future to include the management of retinal vascular diseases and non-uveitic macular edema.

## Introduction

Uveitis is an infectious or non-infectious inflammatory disease of the uvea and other intraocular tissues including the retina, vitreous, and lens. With an incidence of more than 50/100,000-person years, uveitis is a common cause of legal blindness in the western world [1]. In infectious uveitis, the etiology is specific, and the presentation may be characteristic of that infection, although not necessarily for syphilis, tuberculosis, and some AIDS-related opportunistic infections which may mimic many other forms of autoimmune non-infectious uveitis. However, the pathophysiology of non-infectious uveitis is not fully understood, and the etiology might be multifactorial—involving an interplay of genetic, immune, and environmental factors. Viral prodrome that is associated with the presentation of many non-infectious uveitis entities could provide a link connecting the two. Similarly, gut commensals provide a signal directly through the retina-specific T cell receptor and cause autoreactive T cells to trigger uveitis. Microbiota may also serve as an “adjuvant” providing innate signals that amplify and direct the host immune response for development of uveitis [2].

Induction of biologic drugs in the treatment of non-infectious uveitis has introduced a new treatment modality in the field of uveitis. Basic research done in this field has paved way for newer molecules which offer a more targeted and specific therapeutic approach. Anti-TNF-α, considered the first-line biological agent recommended in the treatment of uveitis, has proven remarkably effective. It is by virtue of rapid inflammation control and increased tolerability due to minimal, specific, and manageable side-effects. Despite these benefits, most studies show that approximately 50% of patients with non-infectious uveitis treated with anti-TNF are unresponsive/intolerant [3]. Both physicians and patients have increased expectations from therapeutic agents and end result is not just a good control of inflammation but achieve total clinical remission early in the disease course, so that minimal sequelae result.

Non-responsiveness to TNF blockade and/or residual disease activity, as well as the ongoing, slowly progressing structural damage in a significant proportion of patients treated with TNF inhibitors suggest that—apart from TNF—there are other biological targets involved in the disease process. Interleukin 6 (IL-6) has emerged as a major player in the pathogenesis of autoimmune disease and chronic inflammation. Infection and inflammation cause significant upregulation of IL-6, which features pleiotropic activity and mediates various biological functions. It causes induction of acute-phase reactants in liver, T cell differentiation, regulates inflammatory cells, homeostasis, and healing after tissue injury [4]. Various systemic autoimmune diseases and certain types of cancers have been associated with dysregulation in IL-6 production [5]. In cases of diabetic macular edema, retinal vein occlusion, and chronic uveitis, IL-6 has been found to be significantly elevated in ocular fluids [6, 7]. In the past decades, tocilizumab, a monoclonal antibody (mAB) which targets IL-6 receptor (IL-6R), has been investigated and approved for therapeutic use in a number of immunologic diseases. Several clinical trials have reported it to be effective for the treatment of uveitis and associated macular edema [8–11].

## IL-6 biology: mechanism of action

Human IL-6 is a 26 kDa protein made up of 212 amino acids codified by a gene located in chromosome 7p21 [12]. IL-6 triggers signal transduction after binding the IL-6 receptor (IL-6R). There are two forms of IL-6R: the 80 kDa transmembrane receptor protein and the 55 kDa soluble form (sIL6-R). It is believed that the pleiotropic effect of IL-6 derives from the broad range of cells expressing gp130. IL-6 plays an important role in protecting the host against environmental insults and sends out warning signals through all systems of body about the occurrence of acute events. Physiological levels of IL-6 are very low (1–5 pg/ml) and hardly detectable in serum, but the levels can increase > 100,000-fold during acute inflammatory response [13].

Different cell types in the body produce IL-6, including cells of innate immune system such as neutrophils and monocytes/macrophages. IL-6 is important in the integrated host defense against numerous pathogens including bacteria, fungi, viruses, and mycobacteria [14]. When these infectious pathogens stimulate Toll-like receptors (TLRs) by producing pathogen-associated molecular patterns (PAMPs), they result in prompt production of IL-6 by monocytes and macrophages. In non-infectious inflammation such as during burns and trauma, the affected cells produce damage-associated molecular patterns (DAMPs) which in turn stimulate TLRs, to produce IL-6 [15]. Expression of inflammatory cytokines such as IL-6, TNF-α, and IL-1β is upregulated through various signaling pathways initiated by PAMPs and DAMPs. TNF-α and IL-1β in turn can activate transcription factors to synthesize IL-6 [16].

IL-6 stimulates hepatocytes to induce synthesis of acute-phase reactants like C-reactive protein (CRP), fibrinogen, haptoglobin, alpha-1-antichymotrypsin, and serum amyloid A. CRP is a consistent biomarker of inflammation and is often employed to monitor various inflammatory processes. Its expression mainly depends on IL-6 [17]. Persistent elevation of alpha-1-antichymotrypsin and serum amyloid A has been associated with the pathogenesis of Alzheimer’s disease [18].

Reduced serum iron levels have been reported secondary to IL-6-mediated hepcidin production; latter exerts antagonistic action on the iron transporter ferroportin-1 in intestinal epithelium. This IL-6-hepcidin interaction might be responsible for the anemia found in chronic inflammatory states [19]. IL-6 also upregulates the expression of ZIP 14 (zinc importer), which results in low levels of zinc in chronic inflammation. IL-6 downregulates the expression of fibronectin, albumin, and transferrin. This change in acute-phase reactants is routinely used in clinical laboratory tests for detection of inflammation.

In addition to its role in host defense, IL-6 mediates various biological functions. IL-6 induces activation of stem cells and helps megakaryocytes’ maturation into platelets during hematopoiesis [20]. In bone marrow, IL-6 upregulates the receptor activator of nuclear factor kappa-B ligand (RANKL), which in turn leads to bone resorption and osteoporosis by activating osteoclasts [21].

Production of IL-6 in inflamed tissues upregulates vascular endothelial growth factor (VEGF), which results in increased angiogenesis and vascular permeability [22]. IL-6 promotes proliferation of dermal keratinocytes, and collagen production by fibroblasts, which may contribute to the pathogenesis of diseases like psoriasis, systemic sclerosis, and thyroid eye disease [23].

## IL-6 receptors and signaling pathways

Interleukin 6 plays an important role in host defense against environmental stress such as infection and injury. Dysregulated IL-6 production has been implicated in the development of various autoimmune diseases and chronic inflammatory diseases. IL-6 is a prototypical four-helix bundle cytokine that is a member of the neuropoietins, which includes IL-6, IL-11, IL-27, IL-31, leukemia inhibitory factor, oncostatin M, cardiotrophin-1, neuropoietin, and neurotrophin-1 [19]. These cytokines are structurally related and bind to Class I cytokine receptors. With the exception of IL-31, all IL-6 type cytokines share the membrane glycoprotein gp130 as a common beta receptor and signal transducer subunit [24, 25].

**Fig. 1:**
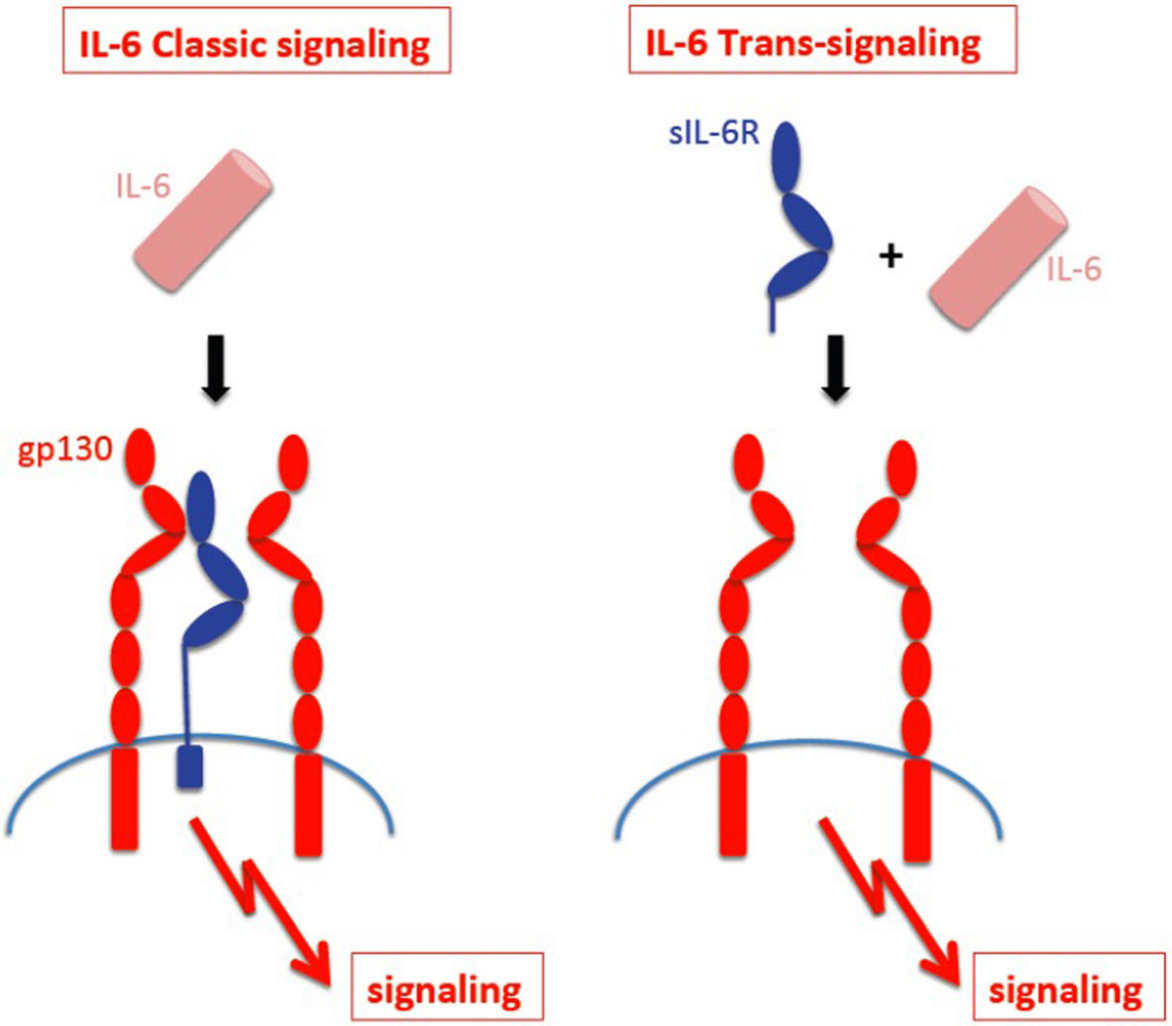
IL-6 classic-signaling and IL-6 trans-signaling. IL-6 classic signaling requires membrane bound IL-6R and is restricted to hepatocytes, some epithelial cells and some leukocytes. IL-6 trans-signaling requires sIL-6R and is possible on all cells of the body since all cells express the gp130 protein. Adapted from “IL-6 trans-signaling via the soluble IL-6 receptor: Importance for the pro-inflammatory activities of IL-6.” by Rose-John S, Int J Biol Sci 2012; 8:1237-1247 [Copyright: Ivyspring International Publisher]

IL-6 signaling occurs through two cellular pathways: the classical and trans-pathway. In the classical signaling pathway, IL-6 binds to membrane-bound type I receptor complex consisting of the ligand-binding glycoprotein, IL-6α. The expression of this receptor is mostly restricted to leukocytes and hepatocytes. The IL-6/IL-6α complex subsequently associates with gp130 leading to gp130-homodimer formation [26]. In trans-pathway, IL-6 provides signaling to cells lacking IL-6R via binding to soluble IL-6R (sIL-6R), which is generated by alternative splicing or ectodomain shedding of the membrane-bound IL-6 receptor (Fig. 1) [27]. Both classical and trans-signaling pathways are gp130-mediated and activate the same intracellular pathways.

After the formation of gp130 homodimer, IL-6 initiates the intracellular signaling by activating the Janus kinase family tyrosine kinases (JAKs) [28]. Activation of these kinases leads to phosphorylation and activation of signal transducers and activators of transcription 3 (STAT3) and the SH2-domain containing protein tyrosine phosphatase-2 (SHP2) [29, 30]. Phosphorylated STAT3 translocates to the nucleus and regulates transcription of various genes. SHP2 activates SOS/Ras-Raf-MEK-MAP kinase pathway to regulate genes [31].

It is important to note that the activation of STAT3 in turn induces the suppressor of cytokine signaling 1 (SOCS1) and SOCS3, which bind tyrosine-phosphorylated JAK and gp130 respectively, to stop IL-6 signaling by means of a negative feedback loop [32, 33].

There is counter-regulation by a soluble form of gp130 (sgp130), present at high concentrations in serum of healthy individuals. As part of the physiological IL-6 buffer in the blood, this natural inhibitor forms a complex with IL-6/sIL-6R, preventing the binding of IL-6/sIL-6R to membrane-bound gp130. This ensures that IL-6/sIL-6R trans-signaling is tightly regulated [34].

**Fig. 2:**
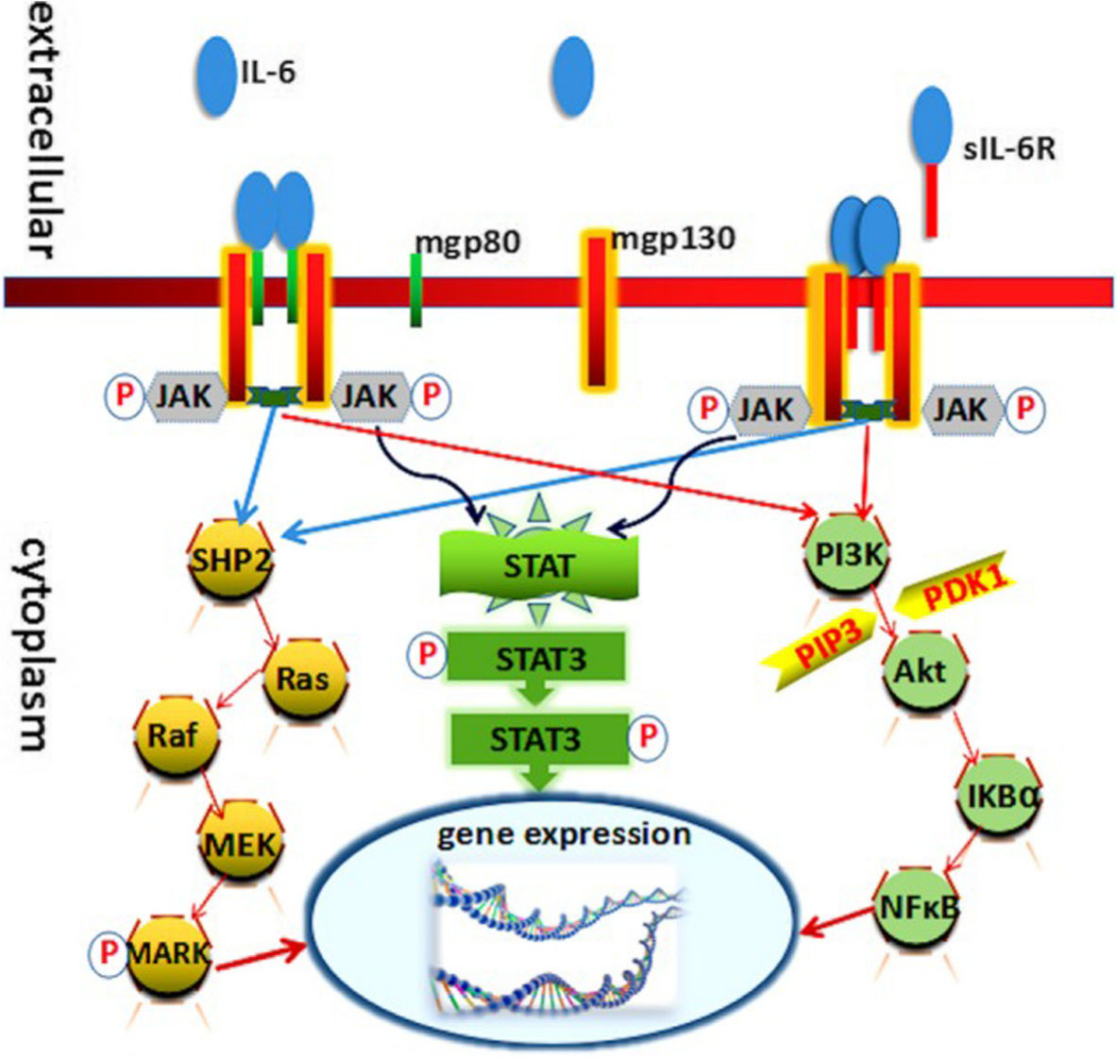
Signaling, activation, and transduction pathways of IL-6. Adapted from “Hall of Fame among Pro-inflammatory Cytokines: Interleukin 6 Gene and Its Transcriptional Regulation Mechanisms.” by Luo Y, Zheng SG. Immunol. 2016; 7:604. [Copyright: © 2016 Luo and Zheng]

Various studies have shown that classic signaling via the membrane-bound receptor is regenerative and protects from bacterial infections, whereas trans-signaling via the soluble receptor is proinflammatory [35]. Therefore, it may make sense to block only the IL-6 trans-signaling alone, which would maintain the regenerative function of IL-6 and specifically suppress only inflammatory arm mediating the disease process (Fig. 2) [27].

### IL-6 and autoimmunity

As mentioned earlier, IL-6 is a pleiotropic cytokine that plays important roles in hematopoiesis, immune defense, and oncogenesis [36]. Historically, IL-6 molecule had been studied under many different names such as B cell stimulatory factor-2 (BSF-2), IFN-β2, Hybridoma/plasmacytoma growth factor, hepatocyte-stimulating factor (HSF), until advancements in molecular testing. In the following sections, we will discuss the role of IL-6 in immune cascades and defense mechanisms, pathological significance of IL-6 signaling in inflammatory autoimmune systemic and ocular diseases, as well as therapeutic implications of IL-6 targeted therapy.

### Immunomodulatory role of IL-6

#### B cells

IL-6 was originally identified and named as B cell stimulatory factor 2 because it promotes the differentiation of activated B cells into plasma cells which are responsible for antibody production [36]. Interactions between T and B cells during antibody production were first reported in 1968 and it was theorized that certain molecules were released from T cells which stimulate B cells to produce antibodies [37]. B cells can produce antibodies, but not without having T cells producing those growth and differentiation factors. IL-6 produced by plasmacytoid dendritic cells is critical for this process [38]. IL-6 also promotes T follicular helper cell differentiation as well as production of IL-21, which also promotes B cell differentiation and increase immunoglobulin synthesis [39, 40].

Moreover, IL-6 may promote the survival of the plasma blasts that secrete immunoglobulin or pathological autoantibodies, e.g., anti-aquaporin 4 in patients with neuromyelitis optica (NMO) [41]. IL-6 may act as an autocrine growth factor in some types of multiple myelomas while some others are themselves able to produce IL-6 [42].

#### T cells

As mentioned, IL-6 was first identified as a B cell function and differentiation factor; however, T cell differentiation and activation is another major action of IL-6 [36]. IL-6 signaling has been found to control proliferation of resting T cells and reinforcing their resistance against apoptosis by inducing IL-2 production and STAT-3 activation [43]. IL-6 has also been identified as major regulator between regulatory T cells (Treg) and effector Th17 cells. In combination with transforming growth factor (TGF)-β, IL-6 brings about differentiation into Th17 cells, but inhibits TGF-β-induced Treg development [44, 45]. This results in an increase of Th17 cell population over Treg cells which may have a role in altered immunological tolerance and resulting in the development of autoimmune inflammatory diseases [46].

IL-6 also modulates Th1 and Th2 balance. It enhances the production of Th2 cells by promoting IL-4 and IL-13 production [47]. On the other hand, it inhibits Th1 cell differentiation and interferon-gamma (IFN-γ) production [48].

### IL-6 and acute-phase response

**Fig. 3:**
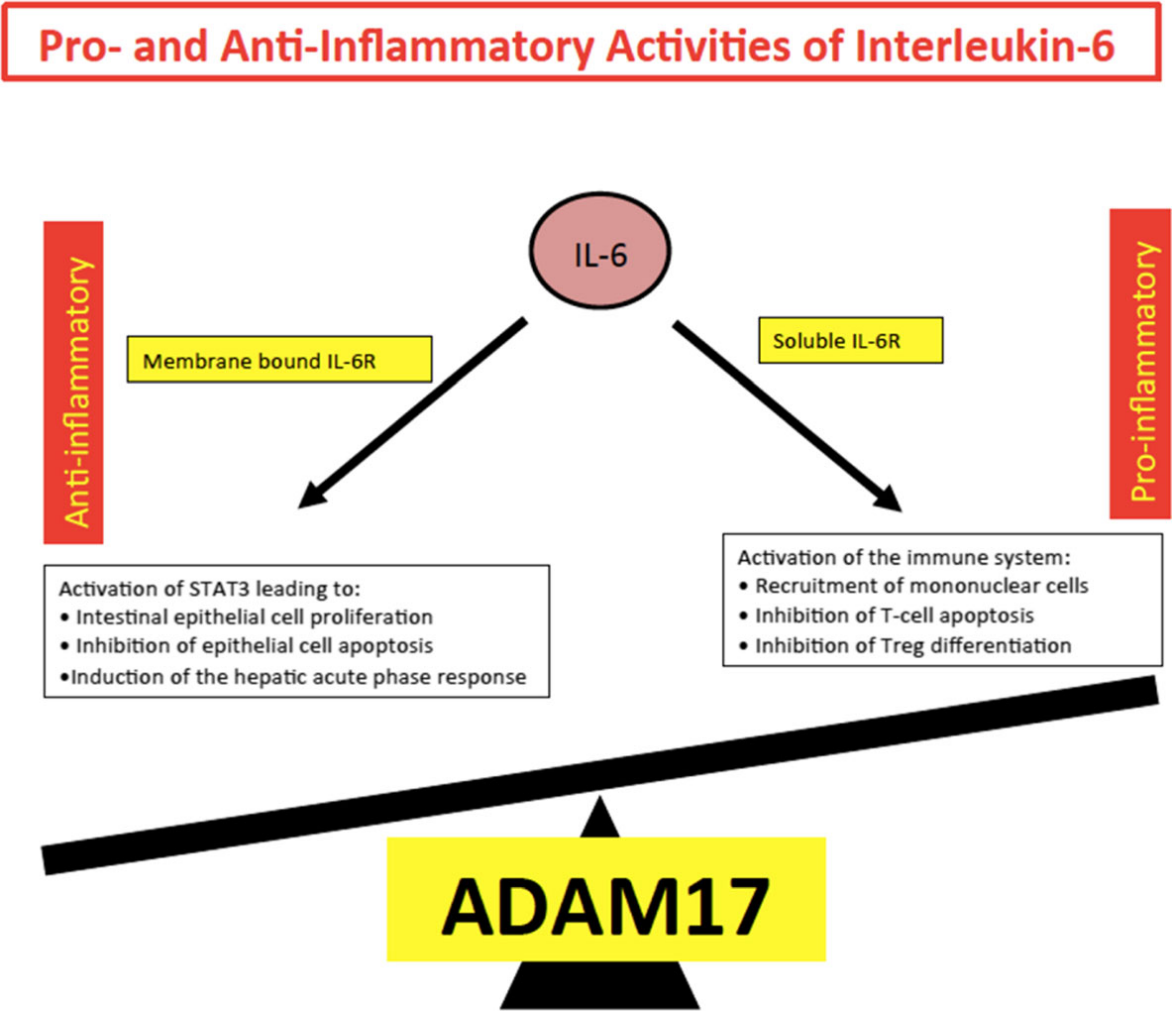
Pro- and anti-inflammatory activities of IL-6. Anti-inflammatory activities of IL-6 include STAT3-dependent regeneration of epithelial cells and the induction of the hepatic acute-phase response. These activities are dependent on the membrane-bound IL-6R. Pro-inflammatory activities of IL-6 include recruitment of inflammatory cells, inhibition of apoptosis of inflammatory cells, and inhibition of regulatory T cell differentiation. Adapted from “Hall of Fame among Pro-inflammatory Cytokines: Interleukin-6 Gene and Its Transcriptional Regulation Mechanisms.” by Luo Y, Zheng SG. Immunol. 2016; 7:604. [Copyright: Ivyspring International Publisher]

IL-6 is a major cytokine in the initiation process of acute-phase responses [36]. In the serum of healthy individuals, the IL-6 level is less than 5 pg/ml; however, IL-6 concentration increases dramatically during infectious and non-infectious events [49]. IL-6 acts as an important factor in the synthesis of acute-phase proteins by the liver, such as C-reactive protein (CRP), serum amyloid A (SAA), fibrinogen, hepcidin, and α1-antichymotrypsin [50]. Administration of IL-6 inhibitors completely normalizes the serum levels of CRP and SAA, indicating that their synthesis depends primarily on IL-6 [51]. These major acute-phase reactants act as an inducer of systemic inflammatory and infectious response. Elevated levels of CRP have been reported in serum of patients with various autoinflammatory diseases such as rheumatoid arthritis, systemic lupus erythematosus, among others, as well as bacterial and viral infections [52]. When the emergent stimuli are completely removed from the host, the IL-6-mediated signal transduction cascade is terminated, leading to normalization of the CRP level in serum (Fig. 3) [53].

### IL-6 regulation

Due to the rapid plasma clearance, IL-6 levels are largely regulated at the transcriptional and post-transcriptional gene expression level [49]. The molecular aspects of IL-6 regulation comprise of complex interactions between proteins, miRNAs, and IL-6 gene expression, which is beyond the scope of this review.

### IL-6 in various systemic autoimmune diseases

IL-6 was first associated with disease development in a case of cardiac myxoma, a benign heart tumor [54]. Subsequently, excessive IL-6 expression patterns were detected in several other autoimmune inflammatory diseases including chronic rheumatoid arthritis, juvenile idiopathic arthritis, systemic lupus erythematosus, Adamantiades-Behcet’s disease, and systemic sclerosis [30, 50, 55, 56]. It is also known that the extent of elevation of serum IL-6 level depends on the type and severity of the disease [57]. On this basis, IL-6 inhibition strategy is currently being pursued to develop novel therapies for inflammatory pathologic conditions. The following sections will focus on IL-6 inhibitors and their usage in systemic and ocular inflammatory diseases.

### Experimental autoimmune uveitis—animal model

Several experimental autoimmune uveitis (EAU) studies demonstrated the importance of IL-6 in non-infectious uveitis (NIU). In an animal model of T cell-mediated uveitis (interphotoreceptor retinoid binding protein (IRBP) immunization model), Yoshimura et al. showed that IL-6-deficient mice could not induce Th17 cells and the EAU score was found to be decreased in those mice in the entire time course [58]. On the other hand, systemic administration of anti-IL-6 receptor antibody reduced uveitic inflammation. This effect in EAU appears to occur via the suppression of both Th1 and Th17 differentiation, both of which are important in this animal model of uveitis. Haruta et al. induced EAU in wild-type (WT) mice and in mice lacking IL-6, IL-17, and IFN-γ and also in IL-6-lacking mice treated with anti-CD 25 monoclonal antibody (mAb) to deplete Treg cells [59]. IL-6 deficiency resulted in the inhibition of the antigen-specific Th1 response and enhanced the generation of antigen-specific Treg cells. Authors concluded that blockade of IL-6 signaling suppresses not only Th17 but also IRBP-specific Th1 by promoting regulatory T cells in EAU.

### IL-6 in ocular pathologies and non-infectious uveitis

IL-6 is a key cytokine which is strongly upregulated during infection/inflammation and associated with variety of systemic autoimmune diseases. Elevated levels of IL-6 have been detected in many ocular diseases such as glaucoma, central vein occlusion, dry eye disease, chemical burn injuries, corneal infections, allergic eye diseases, and ocular inflammatory diseases [56, 60].

Murray et al. was the first to demonstrate elevated aqueous humor levels of IL-6 in 24 human subjects with uveitis, including, Fuchs’ heterochromic iridocyclitis and toxoplasma uveitis [61]. Several groups also found IL-6 to be elevated in ocular fluids including vitreous in patients with active or chronic NIU supporting its mainstream role in ocular inflammatory process [7, 62, 63].

All these evidences, since the first report on the discovery of IL-6 in 1973 by Kishimoto and Ishizaka, have led to a pursuit of new IL-6 inhibitory drugs, for the management of pathologic inflammatory conditions including several types of NIU [64].

Vascular endothelial growth factor (VEGF) plays an important role in the pathogenesis of macular edema in central retinal vein occlusion (CRVO) and diabetic retinopathy by enhancing vascular permeability and altering retinal endothelial tight junctions. In the presence of hypoxia, IL-6 plays a major role in VEGF induction. Vitreous levels of IL-6 and VEGF correlate with the severity of ischemia and are found to be significantly elevated in patients with ischemic CRVO and correlated with the severity of disease [65, 66].

Elevated levels of IL-6 with other cytokines have been shown in proliferative diabetic retinopathy (PDR) wherein the progression may lead to tractional or combined mechanism retinal detachment [67]. IL-6 has also been implicated in the pathogenesis of proliferative vitreo-retinopathy (PVR) after rhegmatogenous retinal detachment (RRD). IL-6 stimulated the production of matrix metalloproteinases (MMPs) which play a major role in the development of PVR [68].

IL-6 has also been found to have a role in various other ocular diseases such as allergic conjunctivitis and dry eye disease, infectious keratitis, ocular neovascularization, and posterior capsular opacification [69–73].

#### Non-specific anti-IL-6 drugs

##### Corticosteroids

The mechanism of action of corticosteroids on IL-6 inhibition is not fully understood; however, they are known to inhibit IL-6 production at the transcriptional level [57, 74]. This process may involve suppression of gene upregulation by nuclear factor-κβ (NF-κβ) or by the occlusion of promotor elements in the IL-6 promotor [5]. Although corticosteroids are the mainstay treatment in many types of inflammatory diseases and IL-6 inhibition is one of their pleiotropic mechanisms of action, their dose-dependent side effects limit long-term therapeutic usage.

These effects are mediated by its specific inhibition of IkB kinase-b, which prevents activation of nuclear factor -kB (NF-kB)

##### Tetracyclines

Tetracyclines are broad-spectrum antibiotics that can act against a wide range of microorganisms via inhibition of protein synthesis [75]. The immunomodulatory and anti-inflammatory properties of tetracyclines suggested that this drug might be effective in the treatment of autoimmune disorders [76]. Wide spectrum anti-inflammatory effects of these drugs are thought to be partially due to suppression of IL-6 by the blockage of NF-κβ signaling [77].

#### Targeted biological anti-IL-6 drugs

##### Tocilizumab

Tocilizumab (Actemra®, Roche AG, Basel, Switzerland) is a humanized mouse monoclonal antibody inhibitor of IL-6 receptor. Tocilizumab (TCZ) prevents the binding of IL-6 with its membrane and soluble receptors and antagonizes its action [4, 6]. It is currently approved for the treatment of rheumatoid arthritis (RA), juvenile idiopathic arthritis (JIA), and giant cell arteritis (GCA) by the FDA [37, 78]. Apart from these, many retrospective open-label studies have shown efficacy of TCZ in inflammatory and/or autoimmune diseases refractory to conventional therapy and/or other biologics which included series of other large-vessel vasculitis (Takayasu’s arteritis), Adamantiades-Behçet’s disease (ABD), adult onset Still’s disease, multicentric Castleman disease (approved in Japan), relapsing polychondritis, Cogan’s disease, inflammatory myositis, and lupus [79].

Large randomized controlled trials (RCTs) on TCZ therapy for RA paved way for its usage in other systemic and ocular inflammatory conditions, since it provided valuable information on the efficacy and side effect profile of the drug. TCZ demonstrated therapeutic potential in moderate to severe active RA patients with inadequate response to methotrexate (MTX), or other conventional disease-modifying antirheumatic drugs (cDMARDs) or biological disease-modifying antirheumatic drugs (bDMARDs) like TNF antagonists [36, 37, 49, 50]. Also, TCZ was found to be better than MTX monotherapy, with rapid improvement in signs and symptoms in patients with active RA, for whom previous treatment with MTX/biological agents had not failed [52]. On the other hand, two phase 3 studies showed that immediate initiation of TCZ with or without MTX in early progressive RA was also more effective and associated with sustained remission and low disease activity, but with a similar safety profile compared with initiation of MTX alone [80, 81]. The results of the phase 4 ADACTA study comparing the efficacy of TCZ 8 mg/kg monotherapy versus anti-TNF-α agent adalimumab monotherapy were also in favor of TCZ in terms of reduction of signs and symptoms of RA, in patients for whom MTX was deemed inappropriate [82].

Successful use of tocilizumab with an acceptable safety profile has been reported in patients with NIU who did not respond to several other treatments. In 2011, Muselier et al. reported the first two cases of refractory uveitis (birdshot chorioretinopathy (BSC) and idiopathic panuveitis) treated by tocilizumab with favorable results and acceptable short-term safety profile [83]. After that report, several case series have demonstrated efficacy of tocilizumab in the treatment of uveitis refractory to anti-TNF agents [8, 84, 85]. Papo et al. in their study with eight consecutive unselected patients with severe and refractory non-infectious uveitis including BD, BSC, and idiopathic cases concluded that tocilizumab was safe and promising [86]. After 8 months of median follow-up, six out of eight patients improved in terms of inflammation control. Mesquida et al. reported on the long-term efficacy and safety of tocilizumab for refractory uveitis-associated macular edema [10]. All their patients were refractory to cDMARD and at least one bDMARD prior to initiation of tocilizumab. Uveitis diagnoses were BSC (*n* = 3), JIA-associated uveitis (*n* = 3), and idiopathic panuveitis (*n* = 1). After a 15-month follow up, no serious adverse events were observed. Mean central foveal thickness improved significantly from 550 to 274 μm at month 12. Visual acuity also significantly improved from 0.67 to 0.4 at month 12. The same group published 24 months results of quiescent uveitis patients with recalcitrant uveitic macular edema (ME), treated with TCZ [9]. Diagnoses included patients with BSC, JIA-associated uveitis, idiopathic panuveitis, sympathetic ophthalmia, and ankylosing spondylitis. Sustained inflammatory remission was maintained in all 12 patients. However, an attempt to withdraw TCZ could only be made in five of them because of systemic disease and perceived high risk of visual loss. All five patients in whom TCZ therapy was withdrawn, ME relapsed within 1 to 3 months after cessation. A re-challenge with TCZ infusions in those patients induced recovery. In the study, tocilizumab was generally well-tolerated except one case of dose-dependent neutropenia and another case of pneumonia [9].

**Fig. 4:**
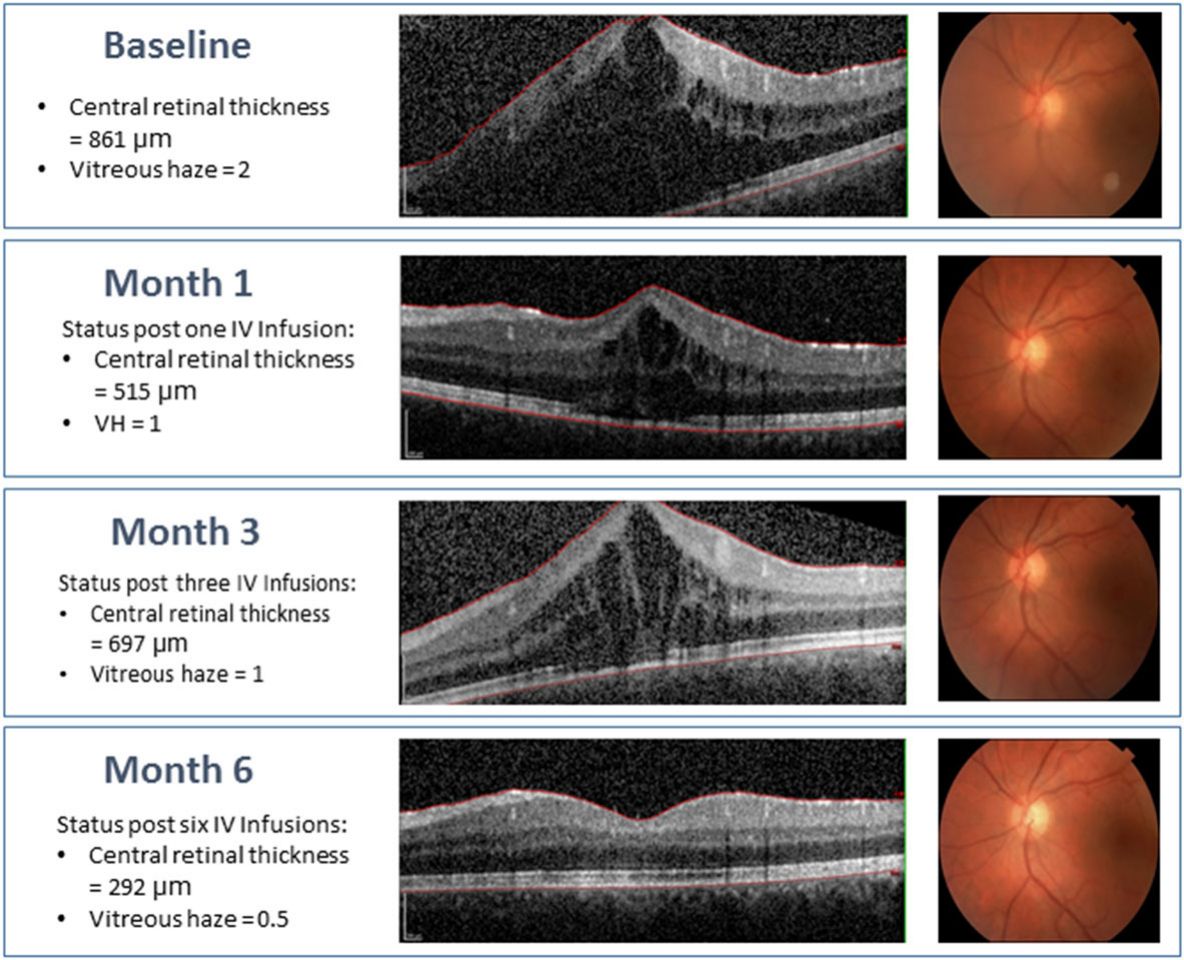
A case of an 18-year-old male subject with idiopathic panuveitis and macular edema who was a subject in the STOP-Uveitis Study, demonstrating changes in vitreous haze and central retinal thickness after treatment with intravenous infusions of 4 mg/kg tocilizumab. The subject was not on any other therapy while he was being treated with tocilizumab. [Adapted from Sepah YJ, Sadiq MA, Chu DS, et al. Primary (month-6) outcomes of the STOP-uveitis study: Evaluating the safety, tolerability, and efficacy of tocilizumab in patients with non-infectious uveitis. *Am J Ophthalmology* 2017; 183:71-80.]

In addition to these encouraging results in small case series and retrospective studies with relatively small number of patients, the first prospective randomized clinical trial STOP-Uveitis was conducted to assess the safety and efficacy of tocilizumab in NIU [11]. STOP-Uveitis was a 6-month study of 37 patients treated with one of two intravenous doses (either 4 or 8 mg per kg) of tocilizumab for posterior NIU. The majority of the cases were of idiopathic origin (28/37 patients) but subjects who had uveitis secondary to Vogt-Koyanagi-Harada syndrome, sarcoidosis, punctate inner choroiditis, and ABD were also included. Only 20–25% of the study population had history of immunomodulatory therapy usage. Two patients developed low absolute neutrophil counts (ANC) after receiving the first infusion of tocilizumab; one normalized before the second infusion while the other subject exited the study. No ocular adverse events related to the study drug were observed. The STOP-Uveitis study demonstrated that the therapy was well-tolerated and associated with a reduction in vitreous haze and cystoid macular edema at both doses (Fig. 4) [11].

##### Tocilizumab: dose and route of administration

The approved starting dose of TCZ differs in various regions of the world. TCZ also has two approved systemic modes of administration: intravenous (IV) and subcutaneous (SC). In the USA, induction therapy employs 4 mg/kg monthly IV regimen followed by step up to 8 mg/kg monthly based on therapeutic response. However, in Europe, the approved initiating and maintenance dose is 8 mg/kg IV monthly, titrated to 4 mg/kg if side effects occur.

SC route of administration has important advantages such as providing more convenient route of administration, including self-application at home, negating the requirement for and associated health care costs of intravenous access, and frequent clinic visits. The efficacy of the SC route over IV route was evaluated in several studies. Two randomized, double-masked, 24-week comparative, phase 3 studies demonstrated the non-inferiority of TCZ-SC to TCZ-IV: SUMMACTA in Europe and MUSASHI in Japan [87–89]. SUMMACTA compared TCZ-SC 162 mg every week to TCZ-IV 8 mg/kg every 4 weeks, and MUSASHI compared TCZ-SC 162 mg every 2 weeks or TCZ-IV 8 mg/kg every 4 weeks, showed safety and non-inferior efficacy of TCZ-SC. BREVACTA, another randomized, double-masked, 24-week comparative trial in the USA, evaluated 162 mg TCZ-SC every 2 weeks versus placebo [88]. BREVACTA showed that TCZ-SC was superior to placebo. These studies led to the approval of the subcutaneous formulation and the dosing recommendations in the European Union and the USA. Consistent with the respective intravenous labels, in the EU, the approved starting dose for TCZ-SC is 162 mg every week, with a possible decrease in dosage frequency to every 2 weeks. In the USA, the starting dose for TCZ-SC is 162 mg every 2 weeks, with a possible increase in dosing to every week, based on clinical response approved [90]. In summary, TCZ-SC appears to be as effective as TCZ-IV with comparable safety.

##### Tocilizumab: safety and pre-administration evaluation

The safety of TCZ in patients with RA has been evaluated in several phase 3 and 4 RCTs [37, 50, 53, 80, 82, 87, 88]. Infections such as nasopharyngitis, upper respiratory tract infections, pneumonia, and cellulitis were the most common. Other major adverse events were gastrointestinal perforations (GIPs), neutropenia, and malignancies. Laboratory test abnormalities were also reported with TCZ therapy, including elevated liver enzyme levels, decreased neutrophil counts, and change in lipid levels.

The real-world studies, in line with the data from RCTs, also found higher incidence of GIPs in patients treated with TCZ than in those treated with other biologics or cDMARDs [91, 92]. For every 1000 patients treated with TCZ per year, between one and two additional GIPs might be expected to occur compared with those treated with other TNF inhibitors [93]. In particular, the risk for lower-intestinal perforations (LIPs) seems to be higher in patients with a history of diverticulitis [91, 92].

Malignancy and neutropenia are two additional concerns for patients receiving TCZ. The risk for malignancy is potentially greater in patients with RA who use immunosuppressive agents. The current hypothesis is that inflammatory activity associated with RA drives the increased lymphoma risk. Additionally, some evidence suggests that patients with RA treated with biologics are at increased risk for malignancy, specifically non-melanoma skin cancers, compared with the general population [94]. Despite these inferences, the analysis of the data from phase 3 trials and long-term extension studies did not demonstrate increased risk for overall or site-specific malignancies above the risk expected in patients with RA. The overall malignancy rate observed in tocilizumab, all-exposure population was higher than that in the general population but was consistent with that expected in the RA population and in the geographic regions studied [16].

Data for neutropenia incidence with TCZ are divergent. De Benedetti et al. reported neutropenia incidences as high as 57% in a cohort of 112 patients with JIA [95]. A dose-dependent effect on neutropenia-onset was observed in RCTs, where the incidence of neutropenia at 8 mg/kg was almost twice as observed at 4 mg/kg [37, 49]. In a retrospective cohort study, TCZ was associated with a higher incidence of neutropenia compared with abatacept and infliximab. However, the increased incidence of neutropenia did not result in a higher risk for severe infections [96]. Also, data from these trials confirm that grade 4 neutropenia (< 0.5 ANC × 109/l) is extremely uncommon.

Adverse events reported in RCTs with TCZ in pediatric age group, treated for JIA, were similar to the adult group and included infections, neutropenia, and abnormalities in liver function test results [95, 97]. Notably, no or very few cases of macrophage activation syndrome (MAS), which resolved, were reported in those studies. On the other hand, a patient registry post-marketing surveillance (PMS) in Japan, which was conducted to investigate the safety and effectiveness of TCZ in real-world clinical settings, reported slightly higher adverse event rates than those reported in clinical trials of TCZ for systemic JIA [98]. This dissimilarity was attributed to the fact that RCTs had excluded patients with concurrent medical or surgical conditions; or concomitant diseases of the nervous, renal, endocrine, or hepatic systems.

All this data from RCTs and cohort studies highlights the need for careful patient selection when treating with anti-IL-6 agents (i.e., exclusion of individuals with previous diverticulitis, exclusion of infections such as tuberculosis, fungal, etc.). Patients should be closely monitored for the development of signs and symptoms of infection during and after treatment. In order to ensure safe use of TCZ in daily practice, physicians and patients should also be aware that, under TCZ, conditions such as GIP, MAS (in pediatrics) may occur with mild symptoms only and careful examination and testing are crucial.

#### Newer IL-6 inhibitors

TCZ was launched as the first biologic drug targeting IL-6 in 2010, which provided a strong alternative to anti-TNF-α agents. Clinical success of TCZ was encouraging, which led pharmaceutical industry to undertake research in discovering other IL-6 blocking pathways. Currently, several IL-6/IL-6R inhibitors are under investigation in various phases of different studies (Table 1). These molecules can be broadly divided into two categories:

**Table 1:** Features of various IL-6 inhibitors—available and investigational, [in RA]

	Target	Structure	Dose and route of administration	Current indications (FDA approved)	Potential indications
Tocilizumab	sIL-6R mIL-6R	Recombinant humanized	4–8 mg/kg IV q4wk 162 mg SC q2wk/q4wk	RA, JIA, GCA	Takayasu’s arteritis, Behçet’s disease, adult onset Still’s disease, multicentric Castleman’s disease (approved in Japan), relapsing polychondritis, Cogan’s disease, inflammatory myositis, lupus, NIU
Sarilumab	sIL-6R mIL-6R	Human mAb	200 mg SC q2wk	RA	RA, NIU
Sirukumab	IL-6	Human mAb	FDA approval denied for RA	n/a	–
Siltuximab	IL-6	Chimeric	12 mg/kg q3wk	Castleman’s disease	Multiple myeloma, currently no studies in NIU
Clazakizumab	IL-6	Humanized mAb	25–200 mg SC q4wk	Not FDA approved	Renal transplant, psoriatic arthritis, RA
Olokizumab	IL-6	Humanized mAb	64 mg SC q2wk/q4wk	Not FDA approved	RA
ALX-0061	sIL-6R mIL-6R	Nanobody (heavy chain-only)	n/a	Not FDA approved	SLE, RA
MEDI 5117	IL-6	Human mAb	n/a	Not FDA approved	–

*RA* rheumatoid arthritis, *JIA* juvenile idiopathic arthritis, *GCA* giant cell arteritis, *NIU* non-infectious uveitis, *SC* subcutaneous, *IV* intravenous, *n*/*a* not available, *qnwk* once every “*n*” number of weeks

Targeting IL-6 with Sirukumab, Siltuximab, Olokizumab, Clazakizumab, and EBI-031

## Targeting IL-6R with Sarilumab and ALX-0061

### Sarilumab

Sarilumab (Kevzara®, Sanofi Genzyme, Regeneron Pharmaceuticals, USA) is a fully human anti-IL-6Rα mAb that blocks both classic and trans-signaling pathway by binding to membrane-bound as well as soluble forms of IL-6Rα, with higher affinity as compared to tocilizumab.

Sarilumab received its USFDA approval in May 2017 for treatment of RA. It is indicated for the treatment of rheumatoid arthritis and is undergoing clinical trials for use in the management of posterior segment non-infectious uveitis. In the SATURN study, in patients with non-infectious uveitis, reductions in vitreous haze and steroid dosing were seen in treated patients. Visual acuity and central macular thickness also improved with a 200 mg dose administered subcutaneously every 2 weeks. This drug targets a specific inflammatory mediator and has been associated with fewer side effects than other available therapies. Neutropenia and elevated alanine amino-transferase levels were reported as adverse events [99].

### Sirukumab

Sirukumab (Plivensia®, Janssen Biologics, Horsham, PA GlaxoSmithKline, UK) is a fully human mAB that binds IL-6. US Food and Drug Administration’s Arthritis Advisory Committee did not recommend sirukumab for the treatment of adults with active rheumatoid arthritis (RA). While the committee unanimously agreed that there is substantial evidence of efficacy of sirukumab for the treatment of these patients, the safety profile is not adequate to support its approval. In phase 3 SIRROUND-D study, the primary adverse events were related to immunosuppression, consistent with those found with other DMARDS; however, there was a trend of increased overall mortality with sirukumab compared with placebo [100]. Out of a total 35 deaths in study patients, 34 occurred in those taking sirukumab. Prominent causes of death were major cardiac events, infection, and malignancy. Increased risk for serious infection was associated with sirukumab; opportunistic infection and tuberculosis were both reported with its use.

### Siltuximab

Siltuximab (Sylvant®) is a chimeric (human-murine) mAB that targets IL-6. It is approved for the treatment of patients with multicentric Castleman’s disease by FDA. It can neutralize the IL-6 effect in a number of human malignancies, reducing cancer-related cachexia and anorexia. In a phase I, open-label study, no dose-related or cumulative toxicity was apparent across all disease indications. A dose of 12 mg/kg every 3 weeks was recommended on the basis of the high response rates in Castleman’s disease and the sustained CRP suppression. Most common drug-related adverse event was thrombocytopenia (25%). Randomized studies are ongoing in Castleman’s disease and multiple myeloma [101]. There are no studies at present to indicate its use in NIU.

### Olokizumab

Olokizumab (R-Pharma, UCB) is a humanized mAB that acts on site-3 of IL-6 and prevents binding of IL-6 to its signaling co-receptor gp130, thereby blocking the assembly of IL-6 signaling complex. It was found effective in a 12-week phase 2b study in RA patients who were refractory to TNF inhibitors. OKZ treatment, at several doses, demonstrated similar efficacy to TCZ across multiple endpoints. Most adverse events were mild or moderate and comparable between OKZ and TCZ treatment groups. OKZ produced significantly greater reductions in DAS28 (CRP) from baseline at week 12 compared with placebo. Reported adverse events were consistent with the safety profile expected of this class of drug without newer safety concerns [102]. No studies at present suggest the use of olokizumab in NIU.

### Clazakizumab

Clazakizumab (Vitaeris®, ALD518, Bothell, USA) is also a humanized anti-IL-6 agent. It showed greater affinity and prolonged half-life in comparison to olokizumab. Clazakizumab proved clinical efficacy by achieving its primary endpoint in treating RA patients, refractory to MTX; however, dose-response effect was lacking. Patients achieved significant improvements in disease activity, including higher rates of remission, as compared with patients receiving only MTX. Clazakizumab treatment groups showed a higher rate of serious adverse events (range 8.3 to 13.6%), compared with 3.3% in MTX group. Pharmacological effects of IL-6 blockade showed consistency with levels of IL-6 measured in laboratory [103]. Clazakizumab also has not been evaluated in the management of NIU.

## Conclusion

### Future of IL-6 inhibition

Various studies in the literature show association of inflammatory disorders like uveitis, retinal vascular occlusions, and diabetic macular edema with increased levels of IL-6. Such data provides a clue to possible area of research and clinical trials targeting these diseases using IL-6 inhibitors. In a pre-clinical study, EAU was treated using intraocular IL-6 locally; this points toward the development of intravitreal IL-6 therapy which can help to avoid associated systemic side-effects.

IL-6 provides an alternative to TNF-α inhibition in the management of NIU. Tocilizumab and secukinumab have shown promising results over established biologics. Various other IL-6 inhibitors are under study and could provide similar therapeutic potentials with added advantages.

It has been recognized that the levels of IL-6 were raised in other ocular vascular diseases such as retinal vein occlusion and diabetic macular edema. There are also cases of NIU with macular edema that responded well to tocilizumab therapy but returned after withdrawal of therapy, only to resolve again when treatment was restarted. Further studies are needed to understand the role of IL-6 inhibitors in non-uveitic macular edema, where anti-VEGF therapy remains the standard of care.

Subcutaneous administration has been evaluated in various studies and has been found non-inferior to IV route. Thus, SC administration can further decrease infusion-related inconvenience and expenditure. Once the newer molecules of IL-6 inhibitors, which target only the trans-cellular pathway, become more established, as we gain experience in their use, complications of IL-6 inhibition such as sepsis can be minimized.

### Perspective

In the current era of pharmacotherapy for uveitis, we can theoretically target TNF, IL-1, IL-12/IL-23, IL-6, IL-17, CTLA4, CD20, etc.—various cytokines that have been identified in the serum and eyes of patients with different types of uveitis. There may be several agents that aim at particular cytokines. As clinicians and scientists gain deeper understanding of the pathophysiology of uveitis, we may be able to target individual cytokines for each disease in each patient, moving toward the goal of precision medicine. As part of future therapeutic plans, patients may undergo biomarker profiling, to be performed on serum and ocular fluids for example, *prior to* the initiation of any therapeutic plans. Based on specific biomarker(s) identified for specific patients, we can employ monotherapy (i.e., IL-6 inhibitor) *or* combination therapy (i.e., IL-1 and IL-6 antagonists or anti-TNF and anti-IL-17 pharmacologic agents) to manage the ocular diseases, being vigilant not to acquire excessive immunosuppression.

In summary, increasing number of studies are showing that IL-6 inhibitors can be very effective biologic agents in the management of NIU. Moreover, additional role of IL-6 inhibition may be realized in the future, possibly opening a newer approach to the management of retinal vascular diseases and non-uveitic macular edema.

## Abbreviations

IL-6: Interleukin 6
IV: Intravenous
SC: Subcutaneous
NIU: Non-infectious uveitis
RVO: Retinal vein occlusion
DME: Diabetic macular edema
VEGF: Vascular endothelial growth factor
TNF: Tumor necrosis factor
mAB: Monoclonal antibody
TLR: Toll-like receptors
PAMPs: Pathogen-associated molecular patterns
DAMPs: Damage-associated molecular patterns
CRP: C-reactive protein
ZIP: Zinc importer
RANKL: Receptor activator of nuclear factor kappa-B ligand
JAK: Janus kinase
STAT3: Signal transducers and activators of transcription 3
NMO: Neuromyelitis optica
TGF: Transforming growth factor
IFN-y: Interferon gamma
EAU: Experimental autoimmune uveitis
PG: Prostaglandin
TCZ: Tocilizumab
OKZ: Olokizumab
RA: Rheumatoid arthritis
SLE: Systemic lupus erythematosus
GCA: Giant cell arteritis
JIA: Juvenile idiopathic arthritis
MTX: Methotrexate
BSCR: Birdshot chorioretinopathy
BD: Behcet’s disease
cDMARDs: Conventional disease-modifying antirheumatic drugs
bDMARDs: Biological disease-modifying antirheumatic drugs
MAS: Macrophage activation syndrome
USFDA: United States Food and Drug Administration
GIP: Gastrointestinal perforation
LIP: Lower intestinal perforation
Treg: T regulatory cells
pg: Picogram
